# Continence Promotion and Successful Aging: The Role of the Multidisciplinary Continence Clinic

**DOI:** 10.3390/geriatrics3040091

**Published:** 2018-12-16

**Authors:** Rhena Yoo, Martha Spencer

**Affiliations:** 1 MD Program, Faculty of Medicine, University of British Columbia, Vancouver, BC, V6T 1Z3, Canada; rhena.yoo@alumni.ubc.ca; 2 Division of Geriatric Medicine, Providence Health Care, Vancouver, BC, V6Z 1Y6, Canada

**Keywords:** incontinence, continence clinic, multidisciplinary, geriatrics

## Abstract

Incontinence is a common yet under-recognized issue that impacts quality of life, especially for older adults in whom there is often a multifactorial etiology. A retrospective chart review was performed on a representative sample of patients seen at our multidisciplinary continence clinic in Vancouver, Canada from January to December 2017 inclusive. Initial assessment was performed by the nurse continence advisor (NCA) or geriatrician depending on the source of referral. The pelvic floor physiotherapist (PFP) could then be consulted based on perceived need. The average age at assessment was 76 years old (range 29–102), with 82% of patients ≥65 years and 27% ≥85 years old. The majority of patients were referred for bladder incontinence (72%), with the remaining patients referred for bowel incontinence (28%) or pessary care (7%). Referrals came from a variety of sources including physicians (62%), nurses (22%), allied health care providers (12%) and self-referral (5%). Multimorbidity was common, with 40% of patients having a Charlson Comorbidity Index ≥6. The same proportion of patients (40%) were on ≥5 prescription medications. Many patients were functionally dependent for either instrumental activities of daily living (52%) or activities of daily living (25%). Non-pharmacologic treatments were commonly recommended, with the majority of patients counselled on lifestyle changes (88%) and taught Kegel exercises (70%). For patients seen by the geriatrician, modifications were made to non-continence medications in 50% of cases and medical comorbidities were optimized in 39% of cases. In terms of pharmacologic therapy, over-the-counter (OTC) medications were initiated in 45% of patients whereas continence-specific prescription medications were started in 17% of patients. A multidisciplinary continence clinic can play an important role in promoting successful aging by assessing and treating medical causes of incontinence in medically complex older adults.

## 1. Introduction

Incontinence is defined as the involuntary loss of urine or feces [1]. It is associated with reduced quality of life [2] as well as significant morbidity. Incontinence has been implicated in increased length of hospital admission and mortality [3], decreased survival in institutionalized elderly [4], and increased risk of falls [5].

Despite its association with negative outcomes, incontinence is under-recognized by both older adult patients and their health care providers who may falsely believe it is a normal, untreatable part of aging [6]. As a result, only about half of older adults with incontinence seek help from a general practitioner [7]. According to a Canadian survey on rates of urinary incontinence, 58% of Canadian seniors report at least one urinary tract symptom [8], and this number is expected to increase with the rapid growth rate of the population aged 65 and older [9]. It is therefore imperative that health care providers be more vigilant in screening for incontinence, especially in older adults.

The multifactorial nature of this geriatric syndrome benefits from assessment and intervention by a multidisciplinary team [10]. The continence clinic at St. Paul’s Hospital in Vancouver, British Columbia opened in August 2016 and is one of only a few such clinics in the country, combining the expertise of a nurse continence advisor (NCA), a pelvic floor physiotherapist (PFP), and a geriatrician. Although there are multidisciplinary continence services in Canada [11], our clinic is unique in its focus on medical causes of incontinence.

The NCA provides in-depth education about non-pharmacologic strategies (lifestyle and behavioral interventions) and provides pessary care and intermittent catheterization instruction. The PFP conducts internal and external exams to assess the strength and tone of the pelvic floor muscles and develops individualized exercise programs based on patient goals. The geriatrician specializes in the care of medically complex patients and assesses the need for pharmacologic treatment.

The clinic sees patients of all ages via several different referral sources (self-referral, physician referral, and allied health referral) but specializes in the care of older adults. Patients are triaged to initial assessment by either the geriatrician, if referred by a physician, or the NCA, if referred by other health care practitioners or self-referral. The geriatrician and the NCA then cross-refer to each other and/or the PFP based on perceived need. At the time of study, PFP referrals could only be made internally by the geriatrician or NCA.

Below, we describe the patient population assessed and treated in this multidisciplinary continence clinic in its first year, as well as interventions provided. As one of the first clinics of its kind in Canada, information from this study will not only help us improve services provided in our clinic but may also inform and encourage those who wish to develop other much-needed continence services in Canada and abroad.

## 2. Materials and Methods

Ethics approval for the study was obtained from the University of British Columbia-Providence Health Care Research Ethics Board, REB Number H18-00990. Permission was obtained from Medical Records at Providence Health Care to access physical charts in the clinic as well as electronic charts on Sunrise Clinical Manager.

To generate the study population for this retrospective chart review, a list of new patients seen by the geriatrician and/or NCA in the continence clinic from January to December 2017 was obtained and sorted in alphabetical order by last name. Since this list included patients from the geriatrician’s general geriatric practice, patients seen by the geriatrician primarily for a non-continence issue were excluded (e.g., reason for referral did not mention continence issue or consultation report did not list continence issue as a problem).

Systematic random sampling was performed by including every third name on the final list. Paper and/or scanned electronic charts were reviewed by one reviewer to collect patient data. Data extracted include demographics, referral information, clinic administrative data, past medical and surgical history, medications, functional history, and assessment and management by the geriatrician and/or NCA. Data was recorded on Excel spreadsheets without personal identifying information and analyzed in Excel using percentages, means, standard deviations, and ranges.

## 3. Results

### 3.1. Demographics

In 2017, 225 new patients were seen for assessment in the continence clinic by the geriatrician and/or NCA. We excluded 47 patients who were seen by the geriatrician primarily for a non-continence issue, leaving 178 patients. After systematic random sampling, 60 patients (34%) were included in the study. Patient demographics for this sample are shown in Table 1.

### 3.2. Comorbidities

Multimorbidity and polypharmacy were common in this sample as shown in Table 2. The Charlson Comorbidity Index was used to summarize multimorbidity, with a point added for each decade after 40 years old (maximum 4 points), as described in the original paper [12]. Other geriatric syndromes were also prevalent in the population including falls (48%) and depression/anxiety (33%). Inadequate information was available about baseline cognitive status.

### 3.3. Referral and Triage

Patient referrals came from a number of different sources including physicians (62%), nurses (22%), allied health (12%), and self-referral (5%). Most physician referrals came from other geriatricians (59%), followed by family physicians (19%) and urologists/urogynecologists (11%). Over half (62%) of patients had already seen another specialist about their bladder/bowel symptoms prior to referral, with 50% of patients who had already been assessed by an urologist/urogynecologist, 13% by a general or colorectal surgeon and 12% by another NCA.

**Figure 1:**
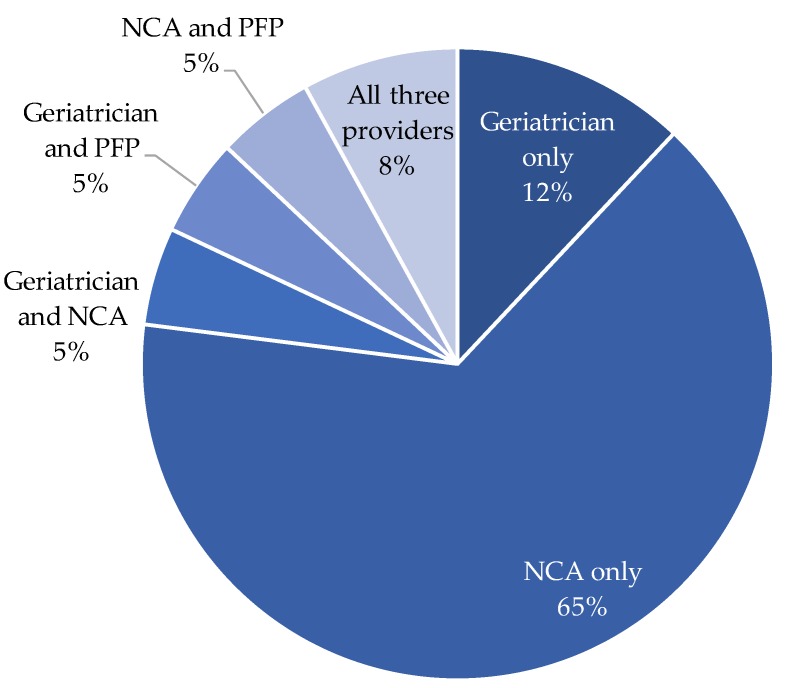
Percent of patients in the study sample seen by one or more providers.

Almost three-quarters of referrals (72%) were for bladder symptoms whereas 28% were for bowel symptoms. For those referred for a urinary complaint, 72% were referred for a broad diagnosis of “urinary incontinence” whereas others were referred for more specific symptoms including frequency (16%), nocturia (14%), urgency or urgency incontinence (12%), recurrent urinary tract infection (5%), and self-catheterization teaching (5%). There were also four patients with vaginal prolapse referred for pessary fitting/maintenance. Bowel-related referrals were for fecal incontinence (59%), constipation (29%), fecal incontinence post-rectal cancer (24%), diet management of poor bowel motility (6%), and fecal urgency (6%). Using the aforementioned triaging process, patients were seen by one or more providers (Figure 1). Most patients were seen within 90 days of the initial referral (83%) with 35% of patients seen within 30 days of the referral date.

### 3.4. Management

Investigations (ordered only for patients seen by the geriatrician) included laboratory investigations (33%), imaging (28%), and referral for urodynamics (17%) or cystoscopy (6%).

**Figure 2:**
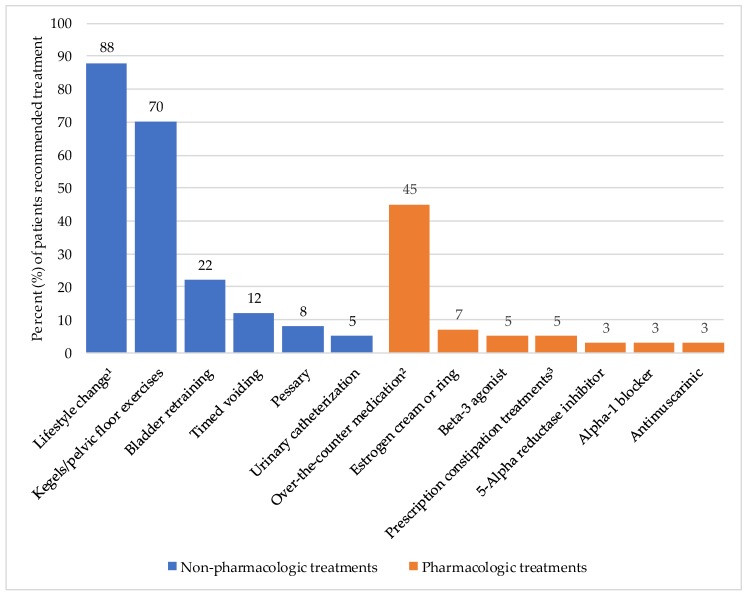
Treatments for incontinence. ^1^ Fluid restriction, increasing water intake, avoiding bladder irritants, dietary modification, increasing fibre intake, exercise and weight loss, personal hygiene education, stress/anxiety reduction techniques. ^2^ Barrier cream, psyllium fibre tablets, polyethylene glycol 3350, senna, bisacodyl, perineal lotion, and non-hormonal moisturizing gel. ^3^ Lactulose, cholestyramine.

Both non-pharmacologic and pharmacologic therapies were used in the treatment of patients with urinary and bowel symptoms (Figure 2). Non-pharmacologic strategies (92%) were used much more commonly than pharmacologic strategies (50%). No data could be provided about treatments provided by the PFP as they were on leave for much of the study period and saw few of the study patients.

With its focus on medical causes of incontinence, the clinic also reviewed and managed non-urinary and bowel issues for 17% of the total sample, with the geriatrician treating such issues for 39% of patients in the sample of patients they saw. Examples of non-continence-related issues assessed and managed include anxiety, back pain, blood pressure, diabetes, osteoporosis, peripheral edema, obstructive sleep apnea, headache, nausea, impaired mobility, functional decline, and caregiver stress. Medications for non-continence-related issues were modified in 50% of patients seen by the geriatrician.

## 4. Discussion

This study aims to characterize the population seen at our novel multi-disciplinary continence clinic and describe a model of care that can be used in the treatment of bladder and bowel symptoms. Male patients made up only 28% of the study sample, which may be explained by the overall lower prevalence of urinary incontinence in men than women [13]. Over 80% of patients in our representative sample were ≥65 years old which is in keeping with the clinic’s specialization in the care of older adults. This is older than the age found at other continence clinics [14,15]. Such clinics specializing in bladder and bowel health in older adults are especially important considering the well-known consequences of incontinence in the elderly including falls [5], social isolation [16] and increased risk of institutionalization [17].

Our continence clinic is unique in its medical, rather than surgical, approach to bladder and bowel issues. Through this chart review, we found that many of our clinic patients had significant multimorbidity, with 40% of patients having a Charlson Comorbidity Index of ≥6. Polypharmacy was also common, with 40% of patients on ≥5 medications. The management of non-continence-related comorbidities and review of non-continence medications by the geriatrician as described in this study is an additional advantage of this clinic for the frail elderly as compared to surgically focused clinics where non-continence-related medical issues might not be reviewed in the same depth.

This study also collected information on the types of cases seen at the continence clinic. Many referrals were received for “urinary incontinence” rather than a specific subtype, which reflects previous work showing that, for community-dwelling older adults, the type of incontinence was diagnosed in less than half of cases [18]. Although the clinic’s initial intention was to primarily be involved in the management of urinary incontinence, this review found that 28% of referrals had bowel symptoms as the reason for referral. The majority of patients had already been assessed for their incontinence, with 58% of patients in the sample who had seen another specialist physician and 12% who had seen a nurse continence advisor. Specialized continence clinics such as ours are therefore an important resource for older adults, especially the frail, who require a more holistic approach to their care.

As per recent Canadian guidelines [19] and international publications on incontinence [20], non-pharmacologic treatments are first-line for most continence issues. Nurse continence advisors play a pivotal role in patient education for non-pharmacologic strategies [21]. In terms of pharmacologic therapies, 78% of patients seen by the geriatrician were started on a continence-related prescription or over-the-counter (OTC) medication and interestingly, 50% had changes made to non-continence-related medications. A complete medication review to deprescribe or modify non-continence-related medications that may have an effect on incontinence should be performed prior initiating continence-modifying medications [20], especially in older adults who have high rates of polypharmacy. A study by Wagg et al. found that the likelihood of reporting treatment emergent adverse effects (TEAE) with fesoterodine increased with the number of coexisting medications in a cohort of patients >65 years old [22]. This same study also found that TEAE increased with the number of concomitant diseases. Almost 40% of patients assessed by the geriatrician also had non-continence-related medical issues addressed as part of the holistic care model of this clinic.

There are some limitations of this chart review. First, the retrospective nature of the study resulted in amalgamation of visits of different lengths, including some patients who were still undergoing assessment at the end of the study period. This may have resulted in some investigations and treatment modalities being excluded from our study. Another limitation was that data on quality of life and degree of bother from incontinence was not consistently recorded and thus outcome data on this important variable could not be clearly delineated for this study. Consistent data on cognitive status in this older population was also lacking, which is important when considering options for pharmacologic treatments. Lastly, objective outcome data was not available for analysis. After the study period, our clinic implemented validated entrance and exit questionnaires as a means of collecting objective outcome data. At the time of our next chart review, we also hope to have more data on the role of our pelvic floor physiotherapist who was off on leave for most of this study period.

## 5. Conclusions

Our continence clinic is unique in its multidisciplinary approach to the assessment and treatment of medical causes of incontinence. This model is particularly beneficial for older adults who have other geriatric syndromes such as frailty, multimorbidity, and/or polypharmacy that may impact continence. Such patients usually have multifactorial causes of incontinence and require individualized care plans which include non-pharmacologic and, sometimes, pharmacologic strategies based on their unique medical, functional, and social needs. Shared-care continence models with both medical and surgical expertise should be developed to ensure that the unique needs of older adults are being adequately met.

The continence clinic addresses complex incontinence in an elderly multi-morbid population by education on non-pharmacologic treatments, prescribing continence-related medications when appropriate, and reviewing the patient as a whole. Such an approach is key in all aspects of geriatric care, and incorporating multidisciplinary continence clinics into geriatric care models is integral in our quest for successful aging.

## Figures and Tables

**Table 1:** Demographics and other characteristics of the study sample.

Variable		Total	Male	Female
Number of patients in sample population		60	17	43
Average age ± SD (years)		76 ± 14	76 ± 12	75 ± 14
Range of age (years)		29–102	57–102	29–98
Number of patients in age group	<65 years old	11 (18%)	2 (12%)	9 (21%)
	65–74 years old	19 (32%)	8 (47%)	11 (26%)
	75–84 years old	14 (23%)	2 (12%)	12 (28%)
	≥85 years old	16 (27%)	5 (29%)	11 (26%)
Cohabitants	Alone	26 (43%)		
	Spouse/partner	25 (42%)		
	Other	7 (12%)		
	Unknown	2 (3%)		
Any community support ^1^	Yes	20 (33%)		
	No	20 (33%)		
	Unknown	20 (33%)		
Type of housing	Independent living ^2^	52 (87%)		
	Assisted living ^3^	7 (12%)		
	Long-term care	0		
Language of communication	English	51 (85%)		
	Other	9 (15%)		
Current cigarette smoking	Yes	3 (5%)		
	No	52 (87%)		
	Unknown	5 (8%)		
Ever gave birth	Yes			38 (88%)
	No			1 (2%)
	Unknown			4 (9%)
Duration of symptoms	<1 year	16 (27%)		
	1 to <5 years	19 (32%)		
	5 to <10 years	4 (7%)		
	≥10 years	8 (13%)		
	Unknown or N/A	13 (22%)		
Activities of daily living	Independent	35 (58%)		
	≥1 dependent	13 (25%)		
	Unknown	10 (17%)		
Instrumental activities of daily living	Independent	20 (33%)		
	≥1 dependent	31 (52%)		
	Unknown	9 (15%)		
Any gait aid used	Yes	32 (53%)		
	No	26 (43%)		
	Unknown	2 (3%)		
Cognitive testing	MMSE ^4^ < 26	10 (17%)		
	MMSE ≥ 26	8 (13%)		
	MoCA ^5^ < 26	9 (15%)		
	MoCA ≥ 26	3 (5%)		
	Unknown	30 (50%)		

^1^ Home support, housekeeper, neighbor, or friend. ^2^ Apartment, condominium, townhouse, detached house, or seniors independent living facility. ^3^ Includes mental health housing. ^4^ MMSE—Mini-Mental Status Exam. ^5^ MoCA—Montreal Cognitive Assessment.

**Table 2:** Comorbidities and medications.

Variable		Number of Patients
Charlson Comorbidity Index	0–2	7 (12%)
	3–5	29 (48%)
	≥6	24 (40%)
Any abdominal surgery	Yes	31 (52%)
	No	23 (38%)
	Unknown	6 (10%)
Any pelvic surgery	Yes	31 (52%)
	No	21 (35%)
	Unknown	8 (13%)
Average number of systemic prescription medications ± SD		4 ± 3
Range of number of systemic prescription medications		0–10
Number of systemic prescription medications	0 to 4	36 (60%)
	≥5	24 (40%)
Class of medications	Centrally-acting medication ^1^	25 (42%)
	ACEi ^2^ or ARB ^3^	21 (35%)
	CCB ^4^	15 (25%)
	Diuretics	13 (22%)
	Opioids	8 (13%)
	Alpha-adrenergic antagonists	4 (7%)
	Anticholinergics	4 (7%)
	NSAIDs ^5^	2 (3%)

^1^ Sedatives, hypnotics, antipsychotics, selective-serotonin reuptake inhibitors, and cholinesterase inhibitors. ^2^ ACEi – Angiotensin-converting enzyme inhibitors. ^3^ ARB – Angiotensin receptor blocker. ^4^ CCB – Calcium channel blocker. ^5^ NSAID – Non-steroidal anti-inflammatory drug.
